# HEPNet: A Knowledge Base Model of Human Energy Pool Network for Predicting the Energy Availability Status of an Individual

**DOI:** 10.1371/journal.pone.0127918

**Published:** 2015-06-08

**Authors:** Abhishek Sengupta, Monendra Grover, Amlan Chakraborty, Sarika Saxena

**Affiliations:** 1 Amity Institute of Biotechnology, Amity University Uttar Pradesh, U.P., India; 2 Centre for Agricultural Bioinformatics (CABin), Indian Agricultural Statistics Research Institute (IASRI), ICAR, New Delhi, India; Laurentian University, CANADA

## Abstract

HEPNet is an electronic representation of metabolic reactions occurring within human cellular organization focusing on inflow and outflow of the energy currency ATP, GTP and other energy associated moieties. The backbone of HEPNet consists of primary bio-molecules such as carbohydrates, proteins and fats which ultimately constitute the chief source for the synthesis and obliteration of energy currencies in a cell. A series of biochemical pathways and reactions constituting the catabolism and anabolism of various metabolites are portrayed through cellular compartmentalization. The depicted pathways function synchronously toward an overarching goal of producing ATP and other energy associated moieties to bring into play a variety of cellular functions. HEPNet is manually curated with raw data from experiments and is also connected to KEGG and Reactome databases. This model has been validated by simulating it with physiological states like fasting, starvation, exercise and disease conditions like glycaemia, uremia and dihydrolipoamide dehydrogenase deficiency (DLDD). The results clearly indicate that ATP is the master regulator under different metabolic conditions and physiological states. The results also highlight that energy currencies play a minor role. However, the moiety creatine phosphate has a unique character, since it is a ready-made source of phosphoryl groups for the rapid synthesis of ATP from ADP. HEPNet provides a framework for further expanding the network diverse age groups of both the sexes, followed by the understanding of energetics in more complex metabolic pathways that are related to human disorders.

## Introduction

**Fig 1:**
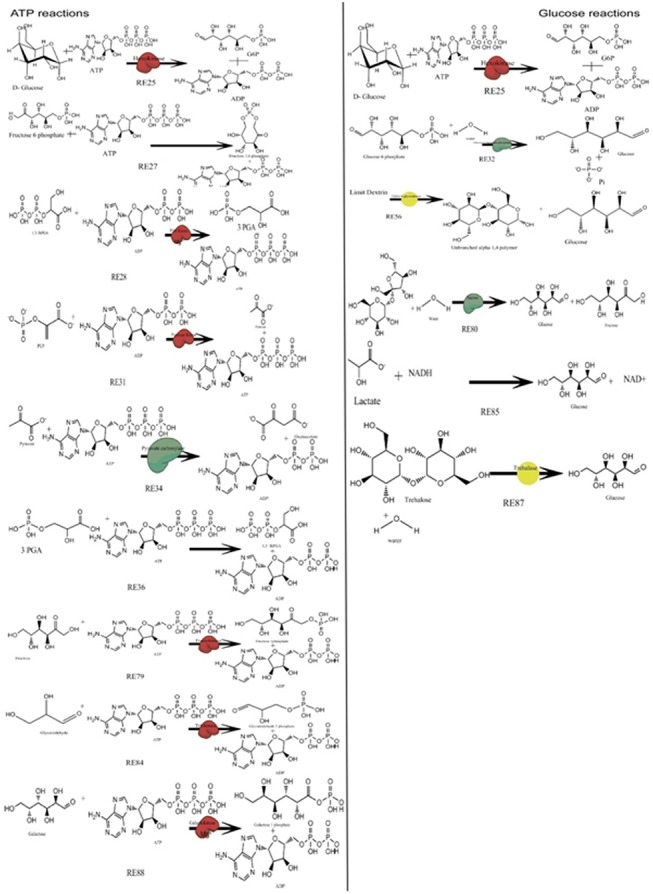
Reactions involving ATP and Glucose used in HEPNet. RE refers to Reaction id. A single ATP molecule is connected to 10 odd reactions and 7 reactions in case of glucose, in the human metabolome. All these reactions are responsible for playing a key role in the Flux Balance Analysis. The cumulative flux of these reactions determines the behavior of HEPNet working in view of Glycaemic condition. As we talk of the energy pool, ATP plays the central role and hence it is reduced in reactions RE25, RE27, RE34, RE36, RE79, RE84, RE88 where reducing agents are Glucose, Fructose 6 phosphate, Pyruvate, 3PGA, Fructose, Glyceraldehyde and Galactose respectively. Also, it has been found that ATP is being produced upon oxidation, where 1,3 BiPGA and PEP acts as oxidizing agents in reactions RE28 and RE31. Glucose is produced in many of the reactions due to the reduction of the product being carried out enzymatically or by the presence of another energy currency NADH.

A comprehensive knowledge of metabolism is fundamental to study and analyze the phenotypic and physiological attributes of all biological system [1]. Normal metabolism is fundamental for well being and an aberrant metabolic condition is apparently a primary cause of many diseases such as diabetes, cancer, neurodegenerative disorders and many more[1]. With the expansion of the high-throughput ‘omics’ technologies, big data illustration into a coherent depiction is a task that has been the research concern of biologists globally. The approach to metabolic reconstruction from the omics data is familiar [2] and its relevance has been well documented. A number of computational and mathematical approaches have been applied to reconstruct the complete metabolic states in humans. The most widely referred and cited one is Recon 1 [3] which broadly represents a comprehensive knowledgebase and has also been reproduced into many useful predictive models [4, 5]. An explicit and rigorous work has also been accomplished on cell type specific reconstructions like HepatoNet1 [6], for intestinal enterocytes [7]. Most of these computational models have been reconstructed from from information derived from genome annotation [8]. Alternatively, constraint based modeling utilizes a mathematical model to analyze the flux rate in the metabolic networks [9]. Mitochondrial centric models have been the major research focus in most of these studies correlating physiological conditions with diseased states. In 2002, Palsson *et al*. presented an excellent study focusing on mitochondrial processes specifically involved in ATP production. This was followed by a large number of studies where metabolic models were reconstructed and used as a device to assess and understand different physiological conditions [10]. Several experimental and computational approaches have been utilized to study the aberrations associated with ATP generation. The metabolic efficiency of any cell is to maintain continuous ATP demand for various processes with the accessibility of substrates including glucose, fatty acids, lactate, pyruvate and amino acids [11]. An illustration of the reactions involving ATP and glucose is portrayed in Fig 1. ATP and other energy currencies like GTP, NADH are vital for a number of important cellular processes that maintain the homeostasis of the human body and therefore it is the need of the hour to model a whole pool where energy currencies play and replay their metabolic roles through anabolism and catabolism. In this paper, we represent a metabolic model of the human energy pool network (HEPNet) using constraint based approach. A hypothesis driven network based on existing knowledge was constructed in the CellDesigner suite. An extensive literature search was undertaken to check the validity of HEPNet, in order to gradients of metabolites under different physiological conditions like glycaemic, uremic and DLDD, physical and feeding states. Evaluation and validation of stress conditions have been monitored by HEPNet with time-course simulation providing an added explanation to the various stress factors given by the Ordinary Differential Equations (ODEs). The completely annotated HEPNet is given in the SBMLfile Model in S2 Dataset. The network establishes a correlation between the identified model patterns through reaction fluxes. We intend to produce an updated HEPNet version 2.0 which could focus on acid lipase disease, Farber’s disease, hyperoxalourea, von Gierke’s disease as well as address conditions of alzheimers, metabolic myopathies, chronic fatigue syndrome. Since, structural features of proteins also play a vital role and the needs a well defined genetic information of the person therefore HEPNet version 2.0 would cater to both genes and metabolites.

## Methodology

### Model Framework

Based on the central hypothesis of energy pool reactions, a comprehensive model of human energy metabolic pool was constructed at the cellular level using CellDesigner suite. On the basis of the information available in the public domain it was postulated to find a consensus of fundamental reactions taking place to maintain and modulate the production of ATP. The metabolic model was built by a bottom-up approach. A complete list of the reactions were assembled together and their mathematical expressions, developed [Table A in S1 Dataset]. The model comprises of 173 metabolic reactions including 4 compartments namely mitochondria, inner mitochondrial membrane, intra-mitochondrial space, outer mitochondrial membrane and 158 metabolites ranging from substrates to enzymes as well as including the energy currencies ATP, GTP, NADH etc. The set of components with description can be found in Table B in S1 Dataset. Based on literature, the species had been created and each reaction was given a shape with reference to BRENDA and kinetic functions defined from the SABIO-RK database. Further species showing multiple reactions have been connected and hence the energy currencies were added accordingly. The species showing multiple reactions are shown in Table C in S1 Dataset. Various enzymes (with their Km values included in Table G in S1 Dataset), carrier proteins and co-factors were also added (Table D in S1 Dataset) All the species used in reconstructing the network have been validated and redundancy has been removed by considering the reaction pathways. A set of abbreviations used in HEPNet has been included in Table F in S1 Dataset.

### Model Annotation

The model was semantically annotated as per the MIRIAM guidelines [12]. To understand and bridge the gap between *in silico* and *in vitro* model layout, a redundant series of model building and hypothesis-driven simulation processes were employed. This was achieved using the Systems Biology Markup Language (SBML) to semantically represent the biochemical reactions in biological models [13]. The use of identifiers and names signifying the same entities can hinder the exchange and comparison of SBML models. This issue has been addressed by MIRIAM, a project to systematize the Minimal Information Requested in the Annotation of biochemical Models. By annotating components with Uniform Resource Identifiers associated with data types from controlled vocabularies, the exchange of models is facilitated by following the guidelines set out by MIRIAM and specific biochemical entities obtained from bioinformatics databases. A complete set of the annotation along with the concentration at steady state condition can be found in Table E in S1 Dataset. The model was annotated semantically with the basis of an adult over 18 years of age of both the genders. The concentration of substrates, metabolites, proteins, enzymes, energy currencies and cofactors (in blood) have been taken from literature. One can find the complete set of concentrations in Table E in S1 Dataset with a set of all the species under various categories segregated in Table J in S1 Dataset.

### Model Validation and Condition Testing

Metabolic constraints affecting various conditions were studied. An extensive exploration was performed for the available *in vivo* concentrations of metabolites in humans through which seven different conditions selected were tested for evaluation of the model. The first parameter that was tested was the glycaemic condition where concentration values were used for hypoglycaemic and hyperglycaemic conditions and variations were observed. The parameter to be tested was the starvation / fasting conditions and the variation in the energy curves were seen. The regulation of energy by the Creatine phosphate stands well demonstrated. Creatine phosphate is known for its energy buffer capability. In the muscle and the brain, Creatine phosphate acts as a rapid reservoir for ATPs and thus a reversible reaction catalyzed by phosphocreatine kinase even restores the excited state to a repository of ADPs. Moreover, the effects on the metabolic regulation during the period of exercise were analyzed. Additionally to check the workability of HEPNet, we studied two disease conditions, which are due to metabolic perturbations namely uremia and Dihydrolipoamide dehydrogenase deficiency(DLDD). The basis of uptake of different parameters was to study the effect on the central energy pool under various physiological and disease conditions. Also the physiological states of obesity, starvation and fasting have been implemented. The complete set of reactions can be found in Table H in S1 Dataset. and condition specific reactions in Table C in S1 Dataset.

## Results and Discussion

### Concept of HEPNet and Model Topology

**Fig 2:**
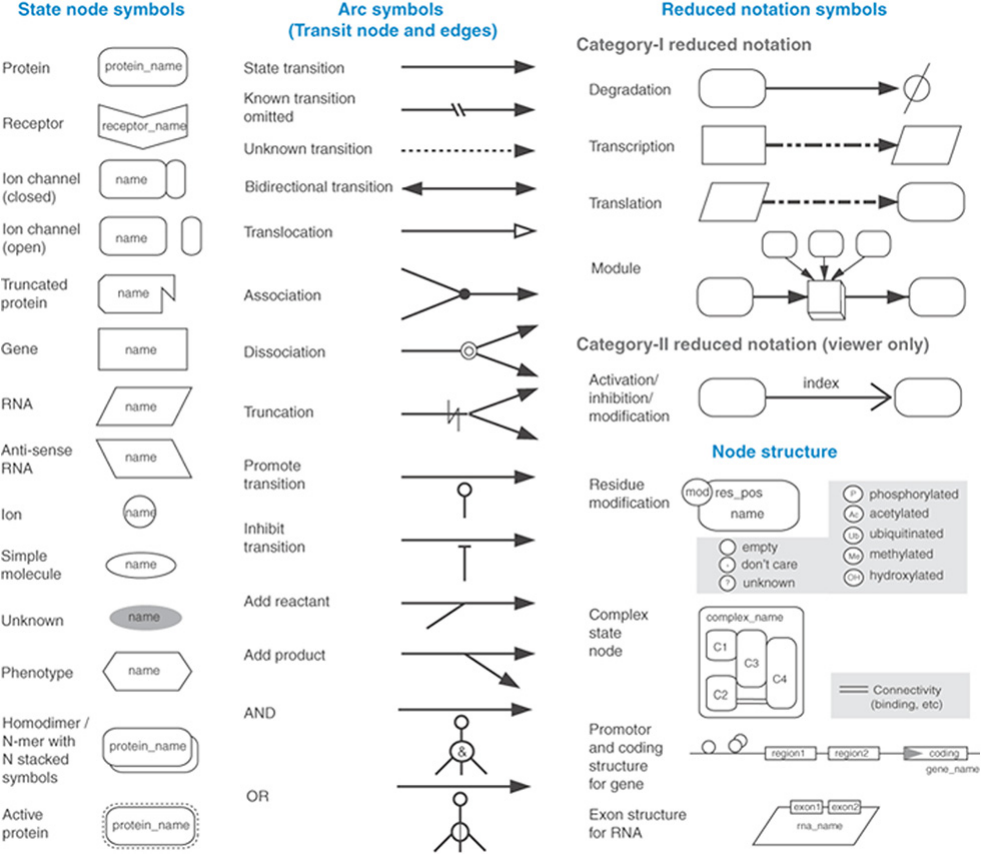
Illustration of the semantically annotated HEPNet. Actual model file is submitted in the SBML file Model in S2 Dataset. Since we are talking of the Human Energy Pool, Mitochondrion is the key organelle. Taylor et al., 2003 [29] said that human mitochondrion is composed of two membranes- outer and inner. In HEPNet, the outer membrane is demarcated in yellow with its inner and outer lining and the inner membrane in magenta with both its inner and outer wall. The reason behind the dual membrane of mitochondrion in HEPNet is to keep up to the actual scenario in a living cell where reactions do take place in the membranes, like transport.

All living cells require constant energy to sustain life processes. There is a constant bartering between demand and supply of energy under normal circumstances. Any system maintains the state of homeostasis i.e. total amount of energy remains constant. HEPNet (Fig 2) depicts the various stages in the form of electronic representation through which energy extraction takes place. HEPNet comprises of simple to complex sugars, fatty acids, monoglycerides, glycerol and amino acids that serve as primary metabolites. Moreover, this network depicts the chemical reactions that convert their primary metabolites into key compounds like acetylCoA which liberate a small amount of energy which can be utilized for maintaining cellular processes. Activities of metabolic pathways are not static but vary in response to the internal and external cues. All the energy generating pathways lead to the production of ATP. The body’s pool of ATP is a small reservoir and is easily accessible. HEPNet, with the help of a visualization platform brings together all these processes involved in ATP production and consumption. HEPNet identifies pyruvate and acetyl CoA as the master regulators of the network which play an important role in several physiological conditions tested as well as linked to diseases. HEPNet consists of a series of chemical reactions that either break down a large compound into smaller units or build up complex molecules.

**Fig 3:**
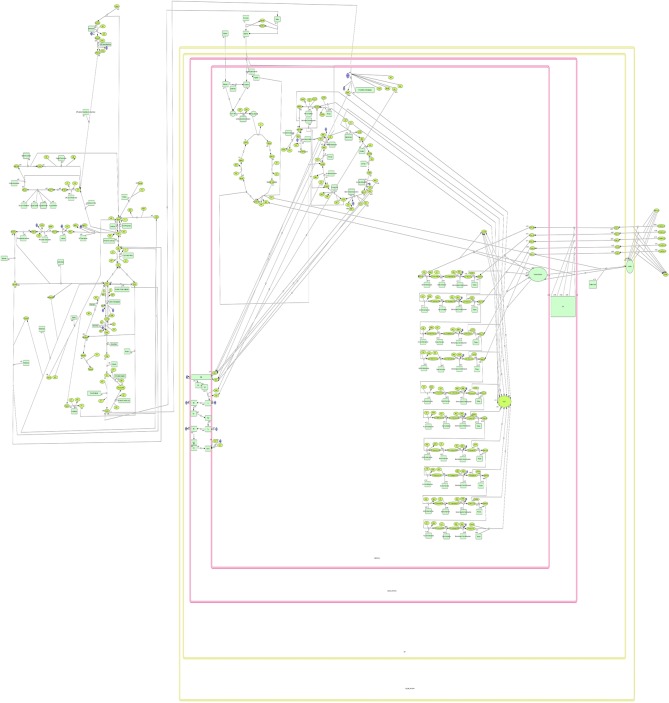
Symbols and Expressions used in CellDesigner by Kitano et al., 2007[21]. Dimensions and type of species in use plays a vital role in designing of complex metabolic networks. CellDesigner has a wide variety of notations to represent the species. There are state node symbols which represent for example a protein or a receptor. Also, there are the arc symbols which are the transit nodes and edges. Further each node has a different node structure depending on the reaction. One advantage of CellDesigner is its view only reduced notation.

The network consists of a total of 173 metabolic reactions including 4 compartments namely mitochondria, inner mitochondrial membrane, intra-mitochondrial space, outer mitochondrial membrane and 158 metabolites ranging from substrates to enzymes. The network model constructed using various components of CellDesigner 4.2 (Fig 3) is annotated with databases ExPASy, KEGG, PubMed, BRENDA to name a few. This network is useful in inferring the behavior of consumption of a specific substrate and the accumulation that occurs during exercise/higher metabolism of the body extending it to starvation or during excess diet. It also bridges the gap and infers the problem of disease diagnosis at a systems level where the main constraint may be due to lower efflux and not any enzyme fault or its inhibition for substrate change or modification; thereby providing an edge over the prevalent Recon 2. Metabolites and enzymes involved in a reaction has been found in databases such as KEGG [14] and Reactome [14], as well as in spreadsheet files that have been used to distribute re-constructed models of metabolism from a number of organisms [15,16]. Enzymes and their kinetic properties are found in various generic and model organism-specific databases which are curated and are included in Uniprot [17], SABIO-RK [18].

A combination of tools is required to model a biological system mathematically [19]. The process begins by mapping the information for each biochemical reaction and its parameters from its source into a model design tool such as CellDesigner [20]. To calibrate parameters by fitting them to a set of experimental observations made from the biological system can then be achieved by biochemical network analysis tools such as COPASI [21] so that a more precise response of the model can be attained in simulations [22, 23]. To understand how biological systems function as a network of biochemical reactions, a redundant series of model building and hypothesis-driven simulation processes are employed. This is achieved using the Systems Biology Markup Language (SBML), a format which is widely used to represent biochemical reactions in biological models [13]. The use of identifiers and names signifying the same entities can hinder the exchange and comparison of SBML models. This issue has been addressed by MIRIAM, a project to systematize the Minimal Information Requested in the Annotation of biochemical Models. By annotating components with Uniform Resource Identifiers associated with conceded data types from controlled vocabularies, the exchange of models is facilitated by following the guidelines set out by MIRIAM and specific biochemical entities referenced by bioinformatics databases [12]. The Systems Biology Results Markup Language (SBRML) specifies quantitative data in the context of a systems biology model [24]. Several databases have been used for the building up of the model which provides insights into literature where the role of any specific metabolite is discussed.

### Model Testing and Functional validation

Time course simulation using COPASI was carried out for validating the different metabolic states and physiological conditions like glycaemic, uremic and DLDD, physical and feeding states and is included in Table B in S2 Dataset. For this purpose Ordinary Differential Equations for each condition has been established and is given in Equations A in S2 Dataset. The solution of the ODEs has been used to validate the actual condition.

#### A. Glycaemic

$$\begin{array}{l} \frac{d[Glu\;\text{c}\;\text{o}\;\text{s}\;e].Vdefault}{dt}=Vdefault\frac{1.[L\;\text{im}\;itD\;extrin]}{\frac{\left(0.08+[L\;im\;itD\;extrin]\right)}{Vdefault}}\\ +Vdefault\frac{1.[Sucrose]}{\frac{\left(10+[Sucrose]\right)}{Vdefault}}+Vdefault\frac{1.[Trehalose]}{\frac{\left(1.37+[Trehalose]\right)}{Vdefault}}\\ +Vdefault\frac{1.[Lactose]}{Vdefault}-Vdefault\frac{1.[Glu\;\text{c}\;\text{o}\;\text{s}\;e]}{\frac{\left(0.048+[Glu\;\text{c}\;\text{o}\;\text{s}\;e]\right)}{Vdefault}}\\ +Vdefault\frac{1.[Lactose][\;N\;A\;D\;H\;]Vdefault}{Vdefault}+Vdefault\frac{1.[G\;6\;P]}{\frac{\left(2+[G\;6\;P]\right)}{Vdefault}} \end{array}$$ (i)

$$Y'=16282.5775-\frac{x}{\left(0.048+x\right)}$$

Integrating w.r.t. x

$$\begin{array}{c} Y(x)=\int (-\frac{x}{0.048+x}+16282.5775)dx\\ Y(x)=16281.6x+0.048\;\text{log}(x+0.048)+C1 \end{array}$$ (ii)

$$\begin{array}{l} \frac{d[ATP].Vdefault}{dt}=Vdefault\frac{1.[3PGA][ATP]}{Vdefault}\\ -Vdefault\frac{1.[Unbranched\alpha 1,4polymer]}{\frac{\left(1+[Unbranched\alpha 1,4polymer]\right)}{Vdefault}}-Vdefault\frac{1.[Fructose]}{\frac{\left(0.58+[Fructose]\right)}{Vdefault}}\\ -Vdefault\frac{1.[Glyceraldehyde]}{\frac{\left(0.31+[Glyceraldehyde]\right)}{Vdefault}}-Vdefault\frac{1.[Galactose]}{\frac{\left(970+[Galactose]\right)}{Vdefault}}\\ -Vdefault\frac{1.[Glucose]}{\frac{\left(0.048+[Glu\;\text{cos}\;e]\right)}{Vdefault}}+Vdefault\frac{1.[PEP]}{\frac{\left(5.8+[PEP]\right)}{Vdefault}}\\ -Vdefault\frac{1.[1,3BiPGA]}{\frac{\left(300+[1,3BiPGA]\right)}{Vdefault}}-Vdefault\frac{1.[Pyruvate]}{\frac{\left(0.11+[Pyruvate]\right)}{Vdefault}}-Vdefault\frac{1.[F6P][ATP]}{Vdefault} \end{array}$$ (iii)

Y'=−57.4x−3.207

-***Glucose factor***

*Glucose factor is that parameter in the equation which is based on the Glucose concentration pertaining to hyper/hypo glycaemic condition. That is if we consider the rate of glucose:

$$-Vdefault\frac{1.[Glu\;\text{cos}\;e]}{\frac{\left(0.048+[Glu\;\text{cos}\;e]\right)}{Vdefault}}$$

We see that it is being consumed as being denoted by the negative sign. Also, irrespective of the default flux value, *Vdefault*, a K_m_ value of 0.048 is responsible. Under steady state condition, the elementary flux modes result can be found in Table A in S2 Dataset, but we may ignore it since all these are constants. What plays the major role is the absolute concentration of glucose at a specific instant. As mentioned in the methodology section, we have considered from literature of hypoglycaemic condition to have a glucose concentration of 400 μM and that of more than 11100 μM in case of hyperglycaemic. Thus, the **Glucose factor** is calculated by using the specific concentration of glucose where the other components of the equation remains constant and do not require to be changed.

**Hypoglycaemic:**

$$\begin{array}{c} Y’=-57.4x-3.207-0.9998\\ Y’=-57.4x-4.2068 \end{array}$$

$$\frac{dy(x)}{dt}=-57.4x-4.2068\;\text{Integrating w}.\text{r}.\text{t}.\;\text{x}$$

$$\begin{array}{c} Y(x)=\int (-57.4x-4.2068)dx\\ Y(x)=-28.7x^{2}-4.207x+C1 \end{array}$$ (iv)

**Hyperglycaemic:**

$$\begin{array}{c} Y'=-57.4x-3.207-0.99999568\\ Y'=\;-57.4x-4.206996 \end{array}$$

$$\frac{dy(x)}{dt}=-57.4x-4.206996\;\text{Integrating w}.\text{r}.\text{t}.\;\text{x}$$

$$\begin{array}{c} Y(x)=\int (-57.4x-4.206996)dx\\ Y(x)=-28.7x^{2}-4.207x+C1 \end{array}$$ (v)

**Fig 4:**
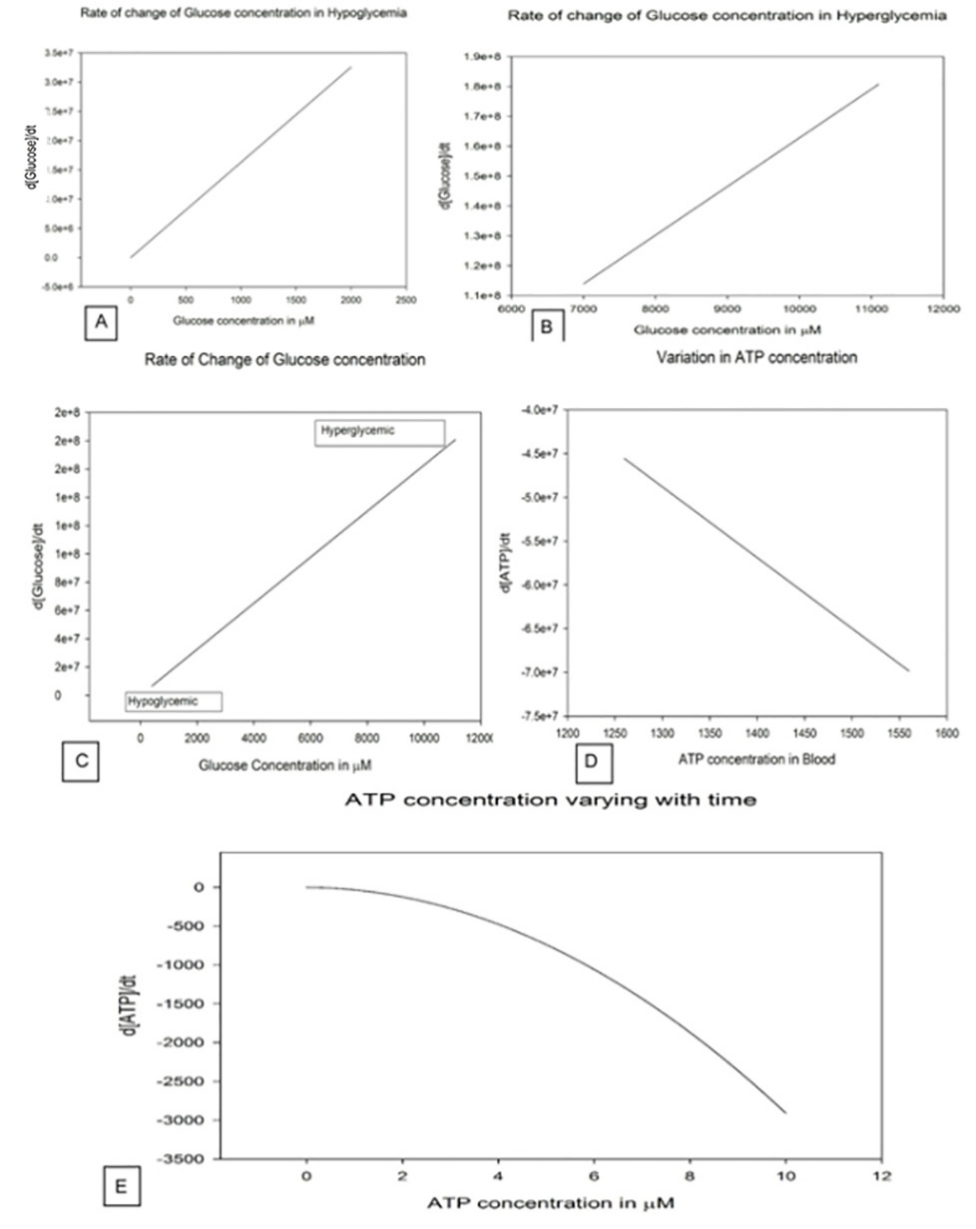
Rate of change of Glucose concentration. **A.** Illustration of rate of change of glucose concentration with respect to the amount of glucose available in hypoglycaemia. d[Glucose]/dt vs. Glucose concentration in μM is plot where a straight line is obtained inferring a gradual increase in the Glucose factor with time. The graph has been made available in the Simulation A file in S2 Dataset. **B.** Illustration of rate of change of glucose concentration with respect to the amount of glucose available in hyperglycaemia. d[Glucose]/dt vs. Glucose concentration in μM is plot where a straight line is obtained but the hyperglycaemic condition starts when the rate of Glucose factor change comes to 1.1e+8 where the Glucose concentration is near 7000 μM. The graph has been made available in the Simulation A file in S2 Dataset. **C.** Illustration of rate of change of glucose concentration with respect to the amount of glucose available, a transition from hypoglycaemia to hyperglycaemia. **D.** Illustrates the decreasing rate of ATP concentration due to consumption with respect to available ATP concentration. **E.** Illustrates the initial drop in rate of ATP concentration as it is compensated by the phosphagen system, glycolysis and krebs cycle.

Comparing the **Glucose factor** in both the two states of glycaemia, it is well understandable that till a precision of 10^–3^ there is no change. But from 10^–5^ there are significant changes. This may sound that at such small levels of precision the equation does not change remarkably. But in actual physiological state, a minute change even in picomolar concentration may lead to severe glycaemic condition as we have can see through Fig 4.

#### B. Starvation

Starvation was evaluated on the basis of the simplified ODE

$$Y(x)=\int \frac{-x}{0.048+x}-1.1586dx$$

The solution to the equation above obtained is

Y(x)=−2.1586+0.048log(x+0.048)+C1

Considering the above equation which is logarithmic and also has an arbitrary constant C1, it is well clear that, a negative factor of -2.1586 plays the role in starvation. This constant value termed as **“Starvation factor”** is derived from the glucose metabolite and plays a key role. Apart from ODE in general, 6 reactions play a major role in starvation but it is the glucose whose limiting nature makes the study important. This distinguishes its role from that of the glycaemic study as we see the difference in both the factors.

#### C. Exercise

Exercise was evaluated on the basis of the simplified ODE

$$Y(x)=\int \frac{-x}{40+x}-0.9976dx$$

The solution to the equation above obtained is

Y(x)=−1.9976+40log(x+40)+C1

Considering the above equation which is logarithmic and also has an arbitrary constant C1, it is well clear that, a negative factor of -1.9976 plays the role in exercise. This constant value termed as **“Exercise factor”** is derived from the acetyl CoA metabolite and plays a key role. Apart from the ODE, in general, 16 reactions play a major role in starvation but it is the acetyl CoA whose limiting nature makes the study important.

#### D. Obesity

Obesity was evaluated on the basis of the simplified ODE

$$Y(x)=\int \frac{-2x}{1+x}dx$$

The solution to the equation above obtained is

Y(x)=−2(x−log(x+1))+C1

Considering the above equation which is logarithmic and also has an arbitrary constant C1, it is well clear that, a product of -2 plays the role in the obesity.

This constant value termed as **“Obesity factor”** is derived from the fact that triglycerides are stored in liver. Triglycerides are generated due to the presence of both glycerol-3-phosphate and fatty acids. The accumulation of triglycerides hence is responsible for obese condition. Only Re82 defines the behavior of the reaction and is responsible for obesity.

#### E. Uremia

**Fig 5:**
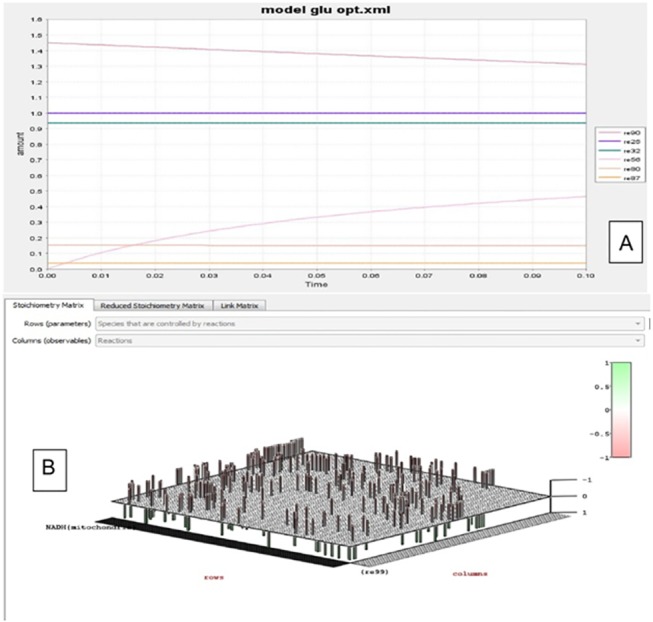
Uremia simulation. A plot showing concentration rates as a function of time which infers that all the reactions tend to obtain a constant rate of reaction as the slope becomes zero.

**Fig 6:**
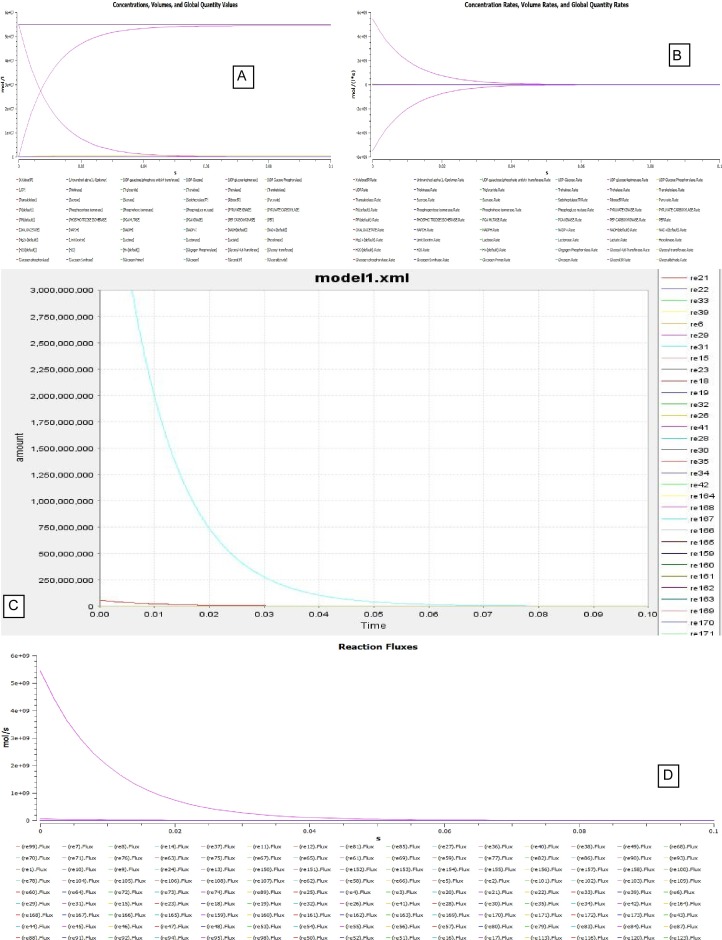
Concentration versus time plot of NADH and NAD^+^. A concentration versus time plot of NADH and NAD^**+**^ depicted together. Green line denotes NAD^**+**^ and blue line denotes concentration change of NADH with respect to time.

**Fig 7:**
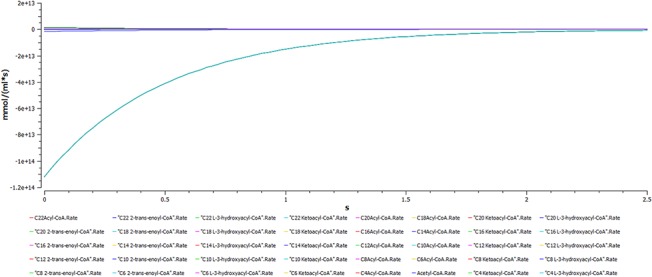
A plot of change in concentrations of C22 trans-enoyl-CoA and C22 L3Hydroxy acyl-CoA. A plot of change in concentrations of C22 trans-enoyl-CoA and C22 L3Hydroxy acyl-CoA over period of time in seconds. C22 trans-enoyl-CoA is denoted in blue and C22 L3Hydroxy acyl-CoA is denoted in green. This is the second step of beta-oxidation after conversion of C22 acetyl-CoA to C22 trans-enoyl-CoA. The concentration of substrate decreases initially very fast but later equilibrium is attained. Similarly the product concentration increases with time.

A plot of dynamic simulation of the constructed model was performed showing comparative time course simulation in CellDesigner and COPASI. A plot showing concentration rates as a function of time was plotted using COPASI. From the graph as shown in Fig 5 it could easily be inferred that reactions tend to attain a constant rate as the slope becomes zero. A concentration versus reaction time of NADH and NAD^+^ was plotted simultaneously as shown in Fig 6 where the green line denotes NAD^+^ and blue line denotes concentration change of NADH with respect to time in seconds. A change in concentrations of C22 trans-enoyl-CoA and C22 L3Hydroxy acyl-CoA as shown in Fig 7 was plotted over period of time. C22 trans-enoyl-CoA is denoted in blue and C22 L3 hydroxy acyl-CoA is denoted in green. This denotes the second step of beta-oxidation after conversion of C22 acetyl-CoA to C22 trans-enoyl-CoA. The concentration of the substrate decreases initially faster but later equilibrium is attained. Similarly the product concentration increases with time.

**Fig 8:**
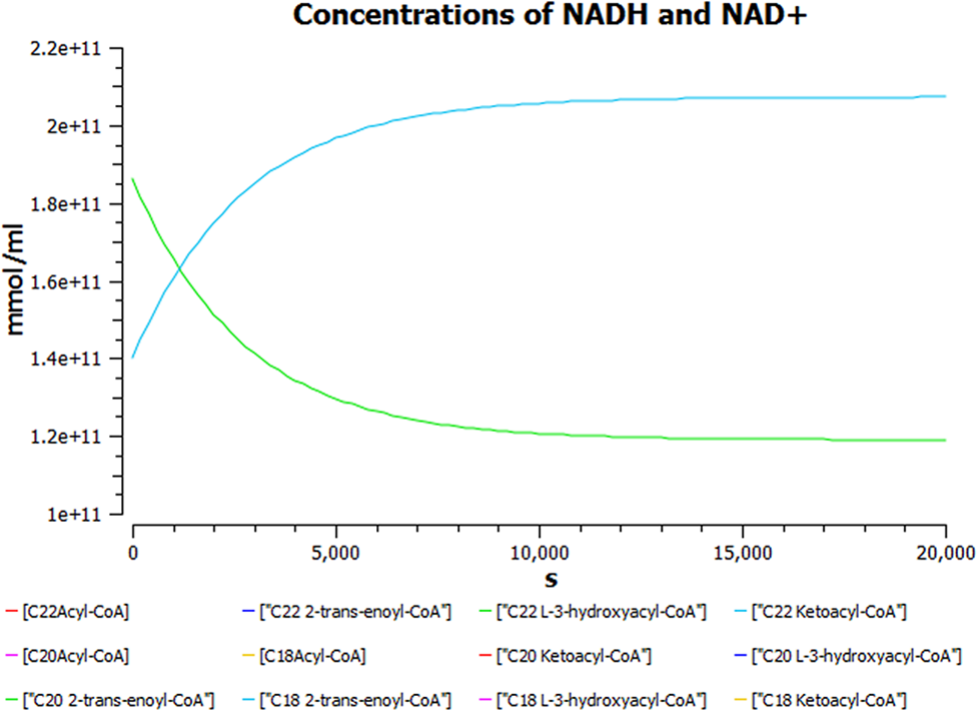
Free carnitine in diseased condition of uremia. **(a):** A 2D Bar chart depicting relative concentrations of Long Chain carnitine and Long Chain acyl-CoA in normal and diseased conditions. **(b):** A 2D Bar chart depicting relative concentrations of Long Chain acyl-CoAs in normal and diseased conditions. **(c):** A 2D Bar chart depicting relative concentrations of Long Chain carnitine in normal and diseased conditions. **(d):** A 2D Bar chart depicting relative concentrations of Long Chain acyl-carnitine and free carnitine in normal and diseased conditions. The results are in accordance with the disease condition of Uremia. **(e):** A 2D Bar chart depicting ratio of relative concentrations of Long Chain acyl-carnitine and free carnitine in normal and diseased conditions. The results are in accordance with the disease condition of Uremia.

Comparative time course simulation plots [Table I in S1 Dataset] generated for the model encompass same plots on performing dynamic simulation of the model using the two separate software programs. This testifies to the reliability of the simulation results as shown in graphs. COPASI program enables the user to choose from a wide range of algorithms, whereas Graphical User Interface of CellDesigner is better. Concentration rates as a function of time were studied. From the graph, plotted in COPASI, it could easily be inferred that all reactions tend to obtain a constant rate of reaction as the slope becomes zero. Time versus concentration plot of NADH reveals that its concentration increases with time, which is in accordance with the published literature. A plot of concentration as a function of time using COPASI output assistant for NAD^+^ in agreement with the concentration increase of NADH depicts that, the concentration of NAD^+^ decreases with time (as shown in plot of NADH with time). Concentrations versus time plot of NADH and NAD^+^ were obtained in a single graph to study their correlation. Green line denotes NAD^+^ and blue line denotes concentration change of NADH with respect to time. The second step of beta-oxidation was studied, after the conversion of C22 acetyl-CoA to C22 trans-enoyl-CoA. A plot of change in concentrations of C22 trans-enoyl-CoA (blue) and C22 L3 hydroxy acyl-CoA (green) over period of time was generated. As a result of dynamic simulation it could be seen that, concentration of substrate decreases initially very fast but later equilibrium is attained and it is also notable that the product concentration increases with time. Scatter plots were generated as well, to gain insights into beta-oxidation of C22 acyl CoA, the reactions were in accordance with classical pathway. A scatter plot of C22 hydroxy acyl-CoA (x-axis) versus C22 trans-enoyl-CoA (y-axis) shows that these two have negative correlation. This is logically validated by the fact that with metabolic cycle progression concentration of C22 Trans-enoyl-CoA decreases whereas that of hydoxy-acyl-CoA increases [25]. Related 2D bar charts were created to study the concentrations of long chain acyl-CoAs, long chain carnitines, free acyl-CoAs, and free carnitine in diseased condition of uremia as shown in Fig 8A–8E [26]. The results depict the increased ratios of long-chain acylcarnitine (LCAC) to free carnitine which is in accordance with the experimental observations [27].

#### F. Dihydrolipoamide Dehydrogenase Deficiency

DLD deficiency is an autosomal recessive metabolic disorder characterized biochemically by a combined deficiency of the branched-chain alpha-keto acid dehydrogenase complex, pyruvate dehydrogenase complex and alpha-ketoglutarate dehydrogenase complex [28].

**Fig 9:**
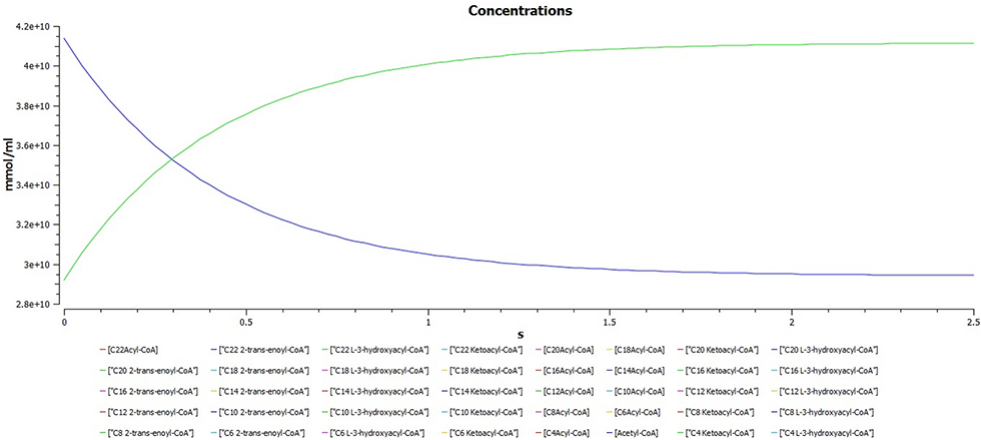
Simulation plot was generated depicting the change of Succynl CoA (SCoA) in TCA cycle with respect to time. **(a)** A plot of change of Succinyl CoA concentration with time for a time period of 1000 seconds **(b)** A plot of Succinyl CoA concentration with time for a time period of 2.5 seconds.

**Fig 10:**
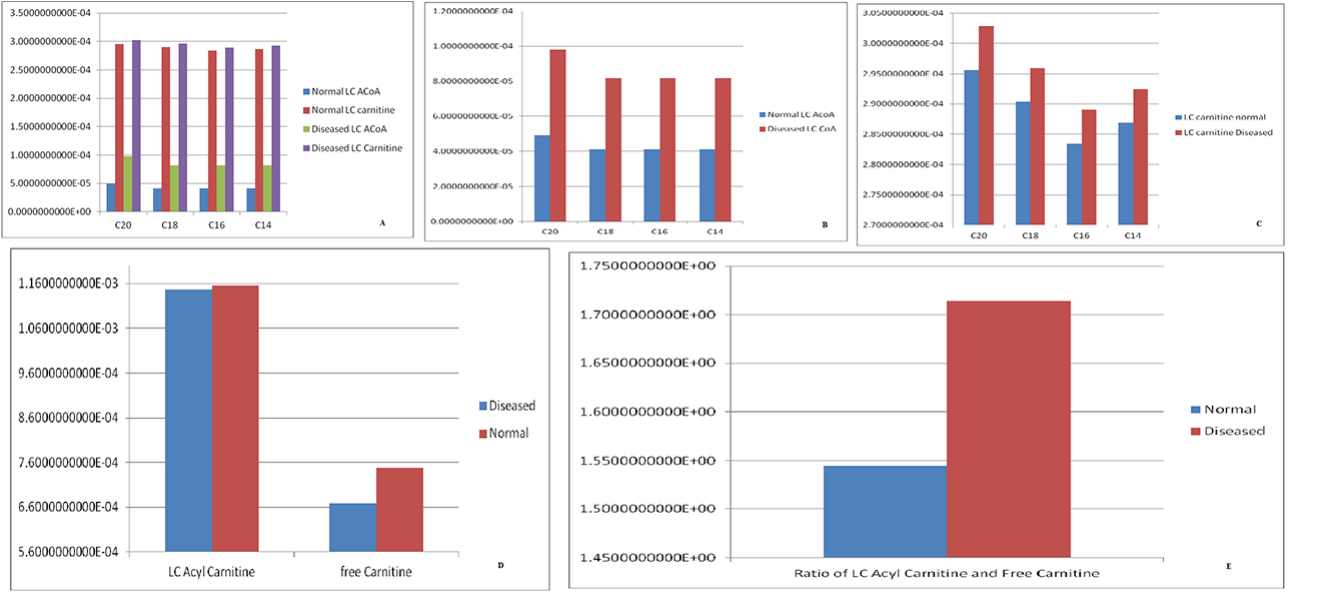
Simulated concentration of alpha-ketoglutarate (AKG) as a function of time. **(a)** Simulated concentration of alpha ketoglutarate as a function of time, increases initially as it is a substrate in rate limiting step of TCA. **(b)** Complete graph showing time course simulation of model for AKG.

**Fig 11:**
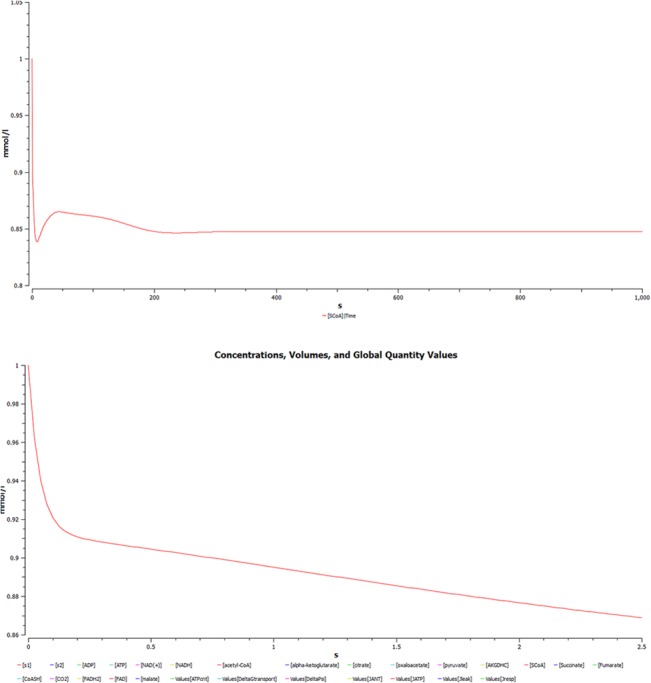
Correlation between NAD+, AKG and NADH. The plots of concentration changes with respect to time between NAD^**+**^ (purple), AKG (blue) and NADH (yellow), this integrated plot shows that rate of change of NADH with NAD^**+**^ and AKG. It clearly depicts that rate of NADH formation depends upon AKG.

(i) **Change of succinyl CoA concentration with time.** A simulation plot was generated depicting the change of succynl CoA (SCoA) in TCA cycle with respect to time as shown in Fig 9A and 9B. A primary graph was plotted with the time period of 1000s. It was observed that concentration of SCoA decreases initially at exponential rate. An increase is seen gradually and the level decreases slightly as the reaction progresses until it acquires a constant rate. To further observe the correlation with time, the time scale of 400 times lesser than the original one gives better insight about the phenomenon. SCoA falls as the initial concentration of SCoA is much more than enzyme concentration then it reaches a plateau phase where SCoA concentration declines even below the critical level.

(ii) **Simulated concentration of alpha-ketoglutarate as a function of time.** A plot of simulated concentration of alpha-ketoglutarate (AKG) as a function of time shows an initial increase, as it acts as a substrate in the rate limiting step as shown in Fig 10(A). Next, a graph is plotted depicting time course simulation of AKG with time as shown in Fig 10(B); since it is the rate limiting step, concentration of AKG increases with time. Gradually as the time progresses, the substrate reaction increases exponentially and the rate of conversion of AKG into SCoA also increases, till it reaches an equilibrium state.

(iii) **Correlation between NAD^+^, AKG and NADH.** The plots of concentration as shown in Fig 11 depicts the changes with respect to time between NAD^+^ (purple), AKG (blue) and NADH (yellow). This integrated plot shows that rate of change of NADH with NAD^+^ and AKG. It clearly shows that rate of NADH formation depends upon AKG.

**Fig 12:**
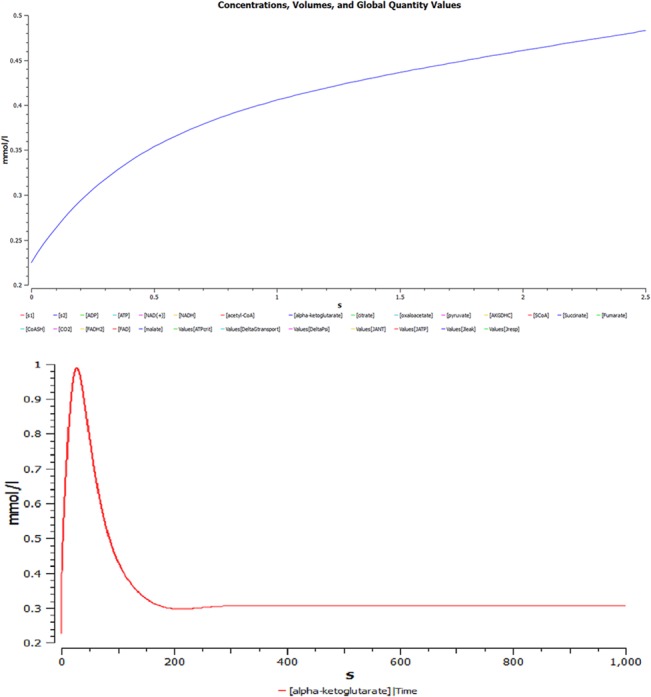
Graph between different concentration of AKGDHC. **(a)** rate of ATP generation with respect to time and **(b)** Pyruvate concentration with time.

Next, a plot as shown in Fig 12(A) for varying concentrations of AKGDHC was generated, and a change in slopes was observed. These plots are of reaction AKG → SCoA with time. With decreasing concentration of enzyme AKGDHC, slope also decreases. Nearly at about 20% inhibition the increase in substrate concentration causes the reaction flux to be maintained to a certain extent. This is because the decrease in enzyme causes increase in AKG concentration which compensates the change in flux, and the AKG level activates the remaining dehydrogenase enzyme. This increase can also be taken as marker for AKGDH impairment as it leads to elevation of alpha- keto glutarate level in blood and urine. The decreasing reaction flux is notable at 40 percent. The flux curve starts to form a straight line at 60 percent inhibition inferring the decreasing reaction flux. Further decrease in slope is visible at 80 percent decrease. At about 95 per cent the reaction flux decreases drastically and it can be assumed that ATP production falls accordingly hence the energy content of cell decreases. The rate of ATP generation with respect to time as depicted in Fig 12(B) shows the drop in level of ATP concentration with decrease in AKGDHC, supports the initial hypothesis that ATP generation is dependent on AKGDHC. A notable diagnostic feature of DLDD where pyruvate is inversely proportional to AKGDHC is illustrated by the plot between the different concentration of AKGDHC and pyruvate.

To understand the effect of reduced AKGDHC on mitochondrial energy metabolism by TCA cycle, a comprehensive SBML model was generated. This model is advancement over the available models of TCA and can be used for studying the effect of AKGDH on the TCA cycle and its substrates. In agreement with the experimental findings, the model simulations confirm a decline in reaction fluxes and NADH level. The finding suggests that it is the rate limiting step of the TCA As has been indicated earlier. Since ATP production is also affected by NADH production rate it can be safely assumed that decrease in NADH also causes change in the rate of ATP production.

Decrease in AKGDH also correlates with loss of glutamatergic neurons as seen in Alzheimer’s and Parkinson’s disease. The simulation suggests a deviations from normal course may lead to the discovery of steps responsible for the disease. Moreover, change in pyruvate concentration on changing the concentration of AKGDH also underpins the importance of the enzymes involved in DLDD. Simulations clearly indicate that AKGDH deficiency may cause increase in pyruvate concentration.

**Fig 13:**
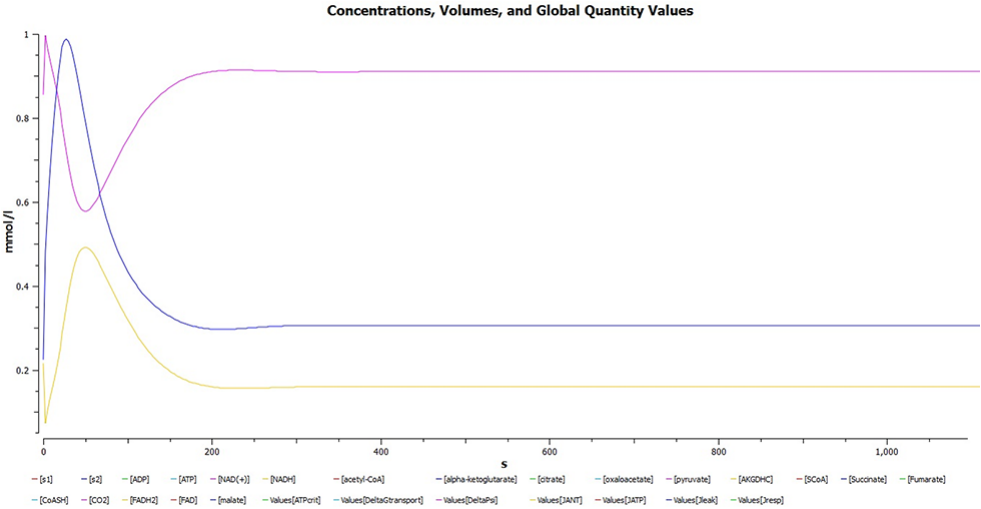
Glucose linked fluxes and Stoichiometric matrix. **A.** 7 reactions of glucose, in the human metabolome representing their fluxes. Glucose is produced in many of the reactions due to the reduction of the product being carried out enzymatically or by the presence of another energy currency NADH in RE85. **B.** Stoichiometric matrix generated using COPASI. Kinetics for multi-compartment reactions are assumed to already be expressed in units of amount of substance (e.g. moles) per time. The resulting value is then multiplied by a factor to convert amount of substance per time to particle numbers per time. Linear combinations of these values, using the stoichiometries as coefficients, result in particle number rates for all species. These form the right hand side of the differential equations.Technically finding the conservation relation means finding rows in the stoichiometry matrix that can be expressed as linear combinations of other rows.

In HEPNet we have made the steady state assumption. The modeled system has entered a steady state, where the concentration of metabolite no longer change, i.e. in each metabolite node the producing and consuming fluxes cancel each other out. The steady-state assumption reduces the system to a set of linear equations, which is then solved to find a flux distribution (Fig 13) that satisfies the steady state condition subject to the stoichiometric constraints while maximizing the value of a pseudo-reaction (the objective function).

**Fig 14:**
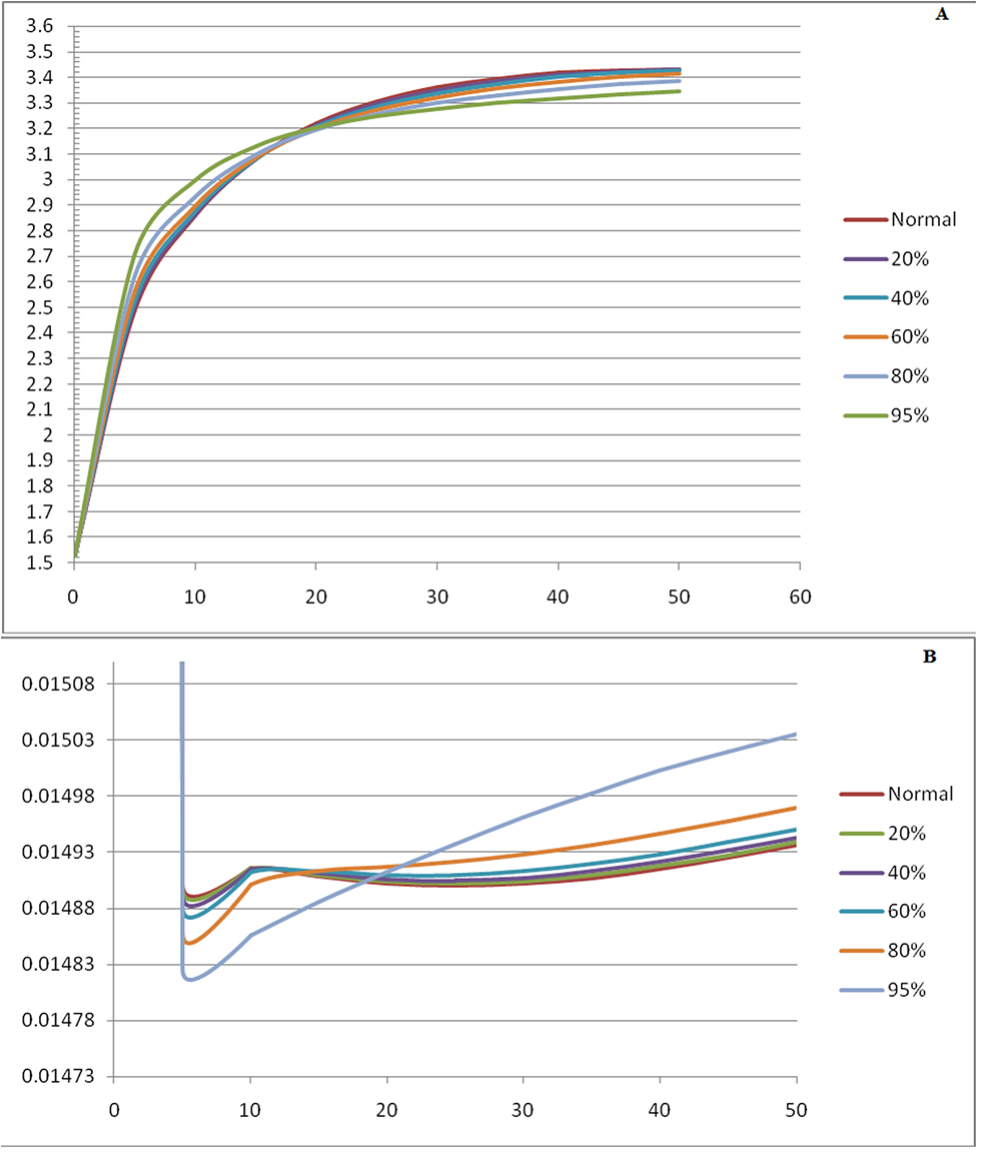
Simulations carried out in COPASI for HEPNet. **A.** Time course simulation of HEPNet using COPASI showing the variation of concentrations in all the reactions under hypoglycaemic condition. A mol/l vs. time curve has been plot, where we can clearly visualize the behavior of exponential rise in ATP concentration to its maximum in 0.06s whereas that of Glucose is decreasing exponentially with time to 0 in about 0.05s. **B.** Time course simulation of HEPNet using COPASI showing the variation of concentrations in all the reactions under Hyperglycaemic condition. A mol/(l*s) vs. time curve has been plot, where we can clearly visualize the behavior of exponential rise in Glucose concentration to its maximum in 0.04s whereas that of ATP is decreasing exponentially with time to 0 in about 0.04s. **C.** Flux generated over time in hypoglycaemic condition of all the reactions in HEPNet using COPASI. The e^**-x**^ plot of mol/s vs. time(s) showing a exponential decrease is due to the flux generated from reactions RE25, RE27, RE34, RE36, RE79, RE84, RE88. The flux becomes 0 in 0.07s. **D**. Flux generated over time in Hyperglycaemic condition of all the reactions in HEPNet using COPASI. The e^**-x**^ plot of mol/s vs. time(s) showing steep exponential decrease in comparison to the hypoglycaemic state which is due to the flux generated from the reactions RE25, RE 28, RE31, RE32, RE56, RE80, RE85, RE87. The flux becomes 0 very quickly in a span of 0.05s which is 0.07s in cance of hypoglycaemic condition.

It is inferable that the flux decreases exponentially in the first 0.02 seconds from Fig 14. In the immediate 0.02 seconds the flux stabilizes to a constant value of 25E+7 (Fig 14) and in a fast span of 0.03 seconds it grounds to zero (Fig 14). A negative slope is observed when the function generated in Eq (iv) is plotted with glucose concentration over a range in hypoglycaemic condition. A negative slope with a straight line thus indicates that the rate of change ATP concentration decreases with increasing ATP concentration (Fig 4D) suggesting the utilization of ATP in the process. It can also be observed from Fig 4E that in steady state mode of operation, the rate of change of ATP concentration is inversely related. Although at the beginning this hypothesis is false since there is a curve. But it stabilizes once the ATP concentration has increased to 8 μM. This behavior of the curve is due to the production of ATP in various processes as well its utilization in the phosphagen, glycolysis and Krebs cycle. Although the solution of ODE presented in Eq (ii) holds a log factor, yet, the graph is a straight line with a positive slope in (Fig 4A–4C). This indicates that the rate of consumption of glucose increases in the body as the concentration of glucose increases from the hypo to hyperglycaemic state.

Several metabolic pathways suffer a setback due to lack of glucose leading to an imbalance in homeostasis although the rate is comparatively low in hypoglycemia.

**Fig 15:**
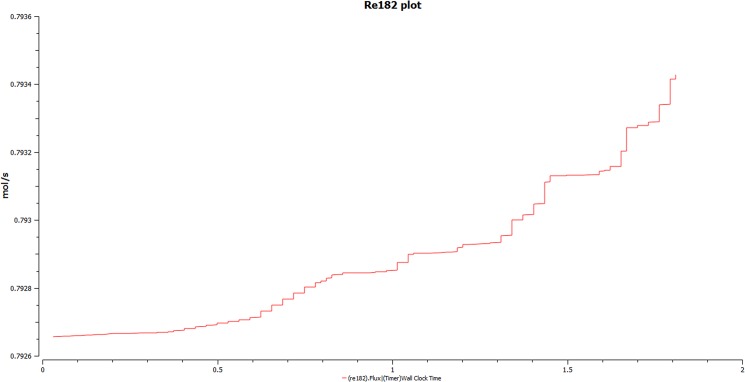
Simulation of the buffering of Creatine phosphate. Reaction 182 demonstrates an exponential steps plot, which signifies the role of creatine phosphate as an energy buffer system. During the need of energy in the system, there is synthesis of ATP and a reversal of the reaction to give ADP by creatine kinase. The buffer-ability of the system is maintained duly by the synergistic role of ATP-ADP and a feedback by the krebs cycle accomplishes the need of the cell for energy.

A remarkable inference to the creatine phosphate can be demonstrated from the flux generated. It is known that, creatine phosphate acts like an ATP reservoir and during the body’s need it gives ATP. Similarly, it also breaks to give creatine and return back ADP where the backward reaction is catalyzed by creatine phosphokinase. Upon observing the flux generated (Fig 15), we observe an exponential step plot. Since, this buffer system is linked to the central metabolic pool of the krebs cycle, where it acts as a control we observe a series of steps. Acting, as a feedback regulation, ATP-ADP acts synergistically to control the flux. Thus, the behavior of a step like curve as observed.

## Conclusion

In terms of an electronic outlook of the representation of HEPNet, manual curation is an added advantage. It can be subjected to plethora of automated simulation to study the effect of ATP generation/loss in a wide range of metabolic diseases and physiological states. For example, the amount of glucose consumed during exercise or starvation could be calculated. All the reactions are compartmentalized and hence the model will be useful in metabolic and metabolomic diagnostics. Its usage is vast and not limited to glycaemia or uremia but even open up dimensions to work upon metabolic biomarkers for the diagnosis of cancer. We envision developing the HEPNet version 2.0 where we intend to use it in case of acid lipase disease (error in fat digestion), Farber’s disease (storage of fats in joints) where a deterministic approach would be necessary. Hyperoxalourea or disease of the urea cycle can even be identified by the faulty flux behavior since HEPNet works on the basis of the energy pool. Another disease named the von Gierke’s disease affecting storage of Type I glycogen could be diagnosed and counter measures can be taken where HEPNet serves as a model for such case, ATP playing a major role.

## Supporting Information

S1 Dataset HEPNet complete reactions, concentrations and conditions data set. Table A_HEPNet complete set of Reactions. Table B_HEPNet compartments. Table C_Condition based reactions. Table D_Classification and complete annotation of all specied used in HEPNet. Table E_concentration references and values of HEPNet. Table F_abbreviations used in HEPNet. Table G_HEPNet species km value chart. Table H_HEPNet complete set of Reactions. Table I_0.1 sec conc variation of subtrates. Table J_HEPNet categorised species.
(ZIP)

S2 Dataset HEPNet Simulation data and actual network. Table A_elementary mode of HEPNet. Equations A_Differential equations of HEPNet. Simulation A_ HEPNet at steady state. Table B_Time course simulation data. Model_HEPNet model in SBML format.
(ZIP)
